# The use of biodegradable temporising matrix for reconstructive salvage following full-thickness chest wall skin graft failure – A case report

**DOI:** 10.1016/j.jpra.2025.08.032

**Published:** 2025-09-02

**Authors:** Harrison Haeata Keane Gregory, Michael Craig Auld, Baillie Ward Churchill Ferris

**Affiliations:** a Department of Surgery, Ipswich Hospital, Chelmsford Avenue, Ipswich, Queensland 4305, Australia; b College of Medicine and Dentistry, James Cook University, 1 James Cook Drive, Douglas, Queensland 4814, Australia

**Keywords:** Biodegradable temporising matrix, Full-thickness skin graft, Basal cell carcinoma

## Abstract

NovoSorb biodegradable temporising matrix (BTM) has emerged as an effective material in the resurfacing of pathologies requiring extensive debridement or tissue loss, such as burns, necrotising soft tissue infections, and tumour excision. The use of BTM in cases of reconstructive salvage following graft failure is less well documented in the literature. We report a case of a 70-year-old female who initially underwent a wide local excision of a large chest wall basal cell carcinoma and full-thickness skin grafting of the resulting defect. Following graft failure and re-debridement, BTM application and consequent split-thickness grafting resulted in a successful reconstructive salvage and cosmetic outcome. This case reinforces the wide-ranging application of BTM in the reconstructive setting, and highlights the effectiveness of BTM in cases of graft failure requiring reconstructive salvage.

## Introduction

Basal cell carcinoma (BCC) is among the most common of skin cancers, accounting for >75 % of malignant lesions and over 900,000 treatments in Australia per year.^1^ They have a predilection for the head, neck and trunk, and are characteristically slow-growing. The mainstay of treatment remains surgical excision, with the aim to maintain cosmesis and functionality. BCCs often reach large sizes as a result of patient neglect, and excision in these settings can cause significant tissue loss when deeper margins are required. Achieving adequate cosmesis can therefore become difficult, requiring the use of skin grafting. In the setting of chest wall lesions, the irregular bony surface of the costosternal junction, relative paucity of subcutaneous tissue, and inherent mobility of the region associated with movements of the pectoral girdle impede the establishment and healing of skin grafts.^2^

NovoSorb biodegradable temporising matrix (BTM; PolyNovo Biomaterials Pty Ltd, Port Melbourne, VIC, Australia) has demonstrated its effectiveness in attaining satisfactory aesthetic results in reconstruction following extensive excision, debridement, or burns. BTM was initially developed in Melbourne in 2004, and since then has seen an increasing utilisation for these indications globally.^3^ BTM is a 2 mm synthetic dermal template composed of a biodegradable polyurethane cell foam, a polyurethane bonding layer, and a superficial non-biodegradable polyurethane sealing membrane.^3^^,^^4^ Typically, BTM achieves wound reconstruction in a two-step process. Initially, BTM acts as a covering and temporising layer to the wound, facilitating new soft-tissue formation via cell migration into the polyurethane foam matrix. Delamination of the non-biodegradable sealing membrane layer then exposes the newly-formed graftable surface. The second step usually involves split thickness skin grafting for definitive wound closure, or alternate methods such as secondary intention healing. The integrated BTM foam layer is completely resorbed over time, primarily by hydrolysis. Overall, BTM provides a dynamic and minimal-risk synthetic substitute for reconstruction in the setting of extensive tissue loss.

## Background

A 70-year-old female presented to the emergency department with a 15-year history of an enlarging and ulcerated skin lesion overlying the sternum measuring 75 × 100 mm. Punch biopsy of the lesion demonstrated a nodular BCC. Preoperative cross-sectional imaging revealed no invasion into the underlying sternum. The patient had no history of melanocyte or keratinocyte skin cancer, immunosuppression, type-2 diabetes mellitus, or other significant medical comorbidities. She was a non-smoker, drank minimal alcohol, and was of normal range BMI.

### Treatment

**Figure 1:**
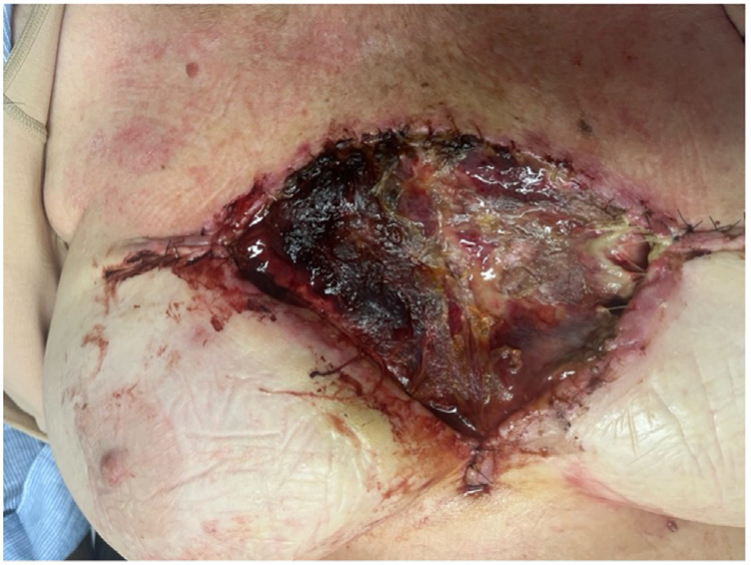
Full-thickness graft failure 12-days following the patient’s index operation.

**Figure 2:**
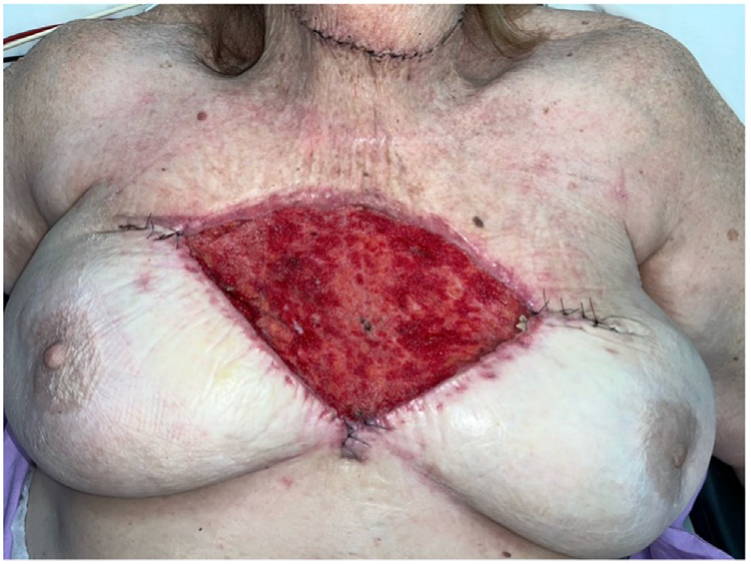
Debridement of the failed full-thickness graft.

The patient underwent wide local excision of her biopsy-proven BCC, with the deep margin taken to the periosteum of the sternum. A full-thickness skin graft was placed over the site of excision during her index operation. The patient was discharged a day following her operation, with a plan for a wound review in the outpatient setting. At 12-days post-operatively, the patient presented to the emergency department with graft failure secondary to wound infection, as seen in Figure 1**.** The patient was placed on intravenous amoxicillin and clavulanic acid, and proceeded to theatre for re-debridement of the wound (Figure 2) and application of a 20 × 10 cm piece of BTM trimmed to the wound area, secured using staples. A vacuum-assisted closure (VAC) device was placed onto the wound intra-operatively.

**Figure 3:**
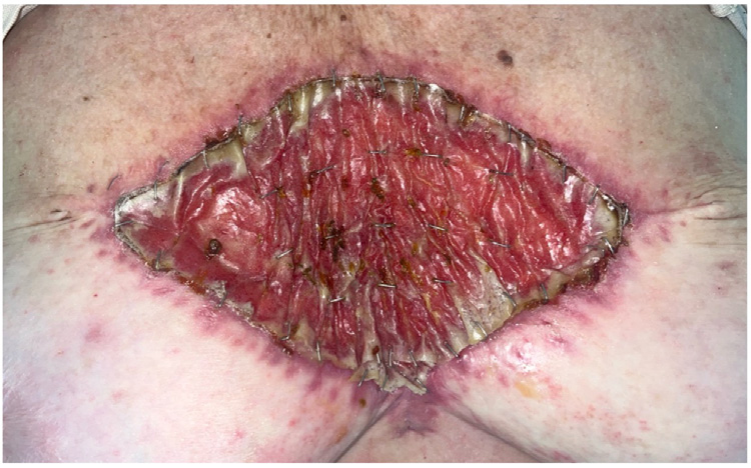
Integrated BTM prior to delamination.

The patient was discharged with a 10-day course of oral amoxicillin and clavulanic acid and had her VAC device removed in the outpatient clinic after 7-days. The VAC device was not replaced, and consequently the wound was dressed with simple dressings. The sealing membrane of the BTM was delaminated after 28-days (Figure 3), and the patient underwent a split-thickness skin graft to the wound bed. The patient then proceeded with once-weekly wound reviews.

### Outcome and follow up

**Figure 4:**
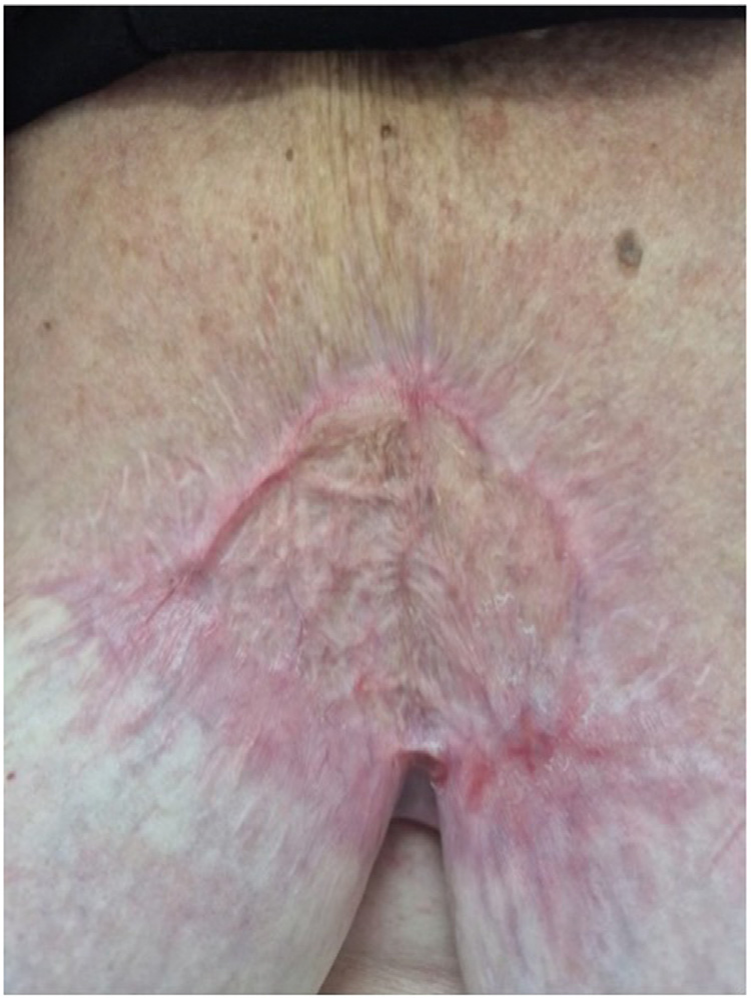
Patient’s wound at discharge.

The patient had one superficial wound infection throughout her follow-up, at 2 months post-BTM application and after skin grafting, which was managed with gentle saline debridement alone. The wound did require periodic administration of topical silver nitrate to areas of hypergranulation tissue. The patient had complete healing of the wound and was discharged from the outpatient clinic approximately 5-months after her initial BCC excision (Figure 4).

## Discussion

Our experience highlights the utility of BTM in achieving salvage wound closure in the setting of skin graft failure. Interestingly there is scant literature concerning the use of BTM for this indication. Generally, BTM has published success rates of up to 90 %, although this rate reflects primary reconstruction rather than salvage reconstruction.^4^, ^5^, ^6^ Given this case, we aim to highlight BTM as a tool for reconstructive salvage.

The mild superficial infection of the wound edge was managed successfully with saline irrigation and simple dressings alone. This required no oral antibiotics. This is opposed to alternative dermal reconstructive substitutes or skin grafting, where superficial wound infection can indicate matrix failure, and necessitate debridement and further operative salvage measures. This highlights a significant benefit of BTM – that despite superficial wound infection, ongoing granulation can proceed without significant patient morbidity. This is supported by a systematic review by Fruergaard et al.^6^ – in 69 studies detailing BTM usage in reconstruction, the overall infection rate was 16 %. Despite this, only 3 % of these patients required removal of BTM following infection.^6^ A systematic review of a similar matrix product, Integra, reported 16.9 % of wounds experienced infection resulting in matrix loss.^7^ This data supports the superiority of BTM in resistance to wound infection.

This case was the first use of BTM at our centre, and as such we have limited experience in the care of patients with BTM. Timelines for delamination, the use of VAC devices, dressings and other logistics are still being investigated – in fact, a strong recommendation for time to delamination are not present in the literature. This timing does depend on the progress of cellular infiltration and integration into the BTM and can vary by patient and wound type. The actual timing of when delamination occurs after integration has been achieved can also vary depending on when the second stage for achieving definitive wound closure is wanted by the surgeon, allowing for some flexibility. This, as well as larger studies exploring the utility of BTM in settings of graft failure, is desirable.

## Limitations

This case study reports on the use of BTM in a single patient with full-thickness skin graft failure. A larger population of patients requiring BTM salvage reconstruction would be beneficial to compare patient outcomes and varying surgical techniques and approach to follow-up.

## Conclusion

BTM is an effective material in the primary resurfacing of wounds resultant from extensive debridement or tissue loss. Although few cases exist in the literature, as demonstrated by this case BTM is a favourable option in cases of reconstructive salvage, and more specifically in cases of resected skin malignancy. The resistance of BTM to wound infection, and its ability to continue integration in the presence of superficial infection is beneficial in these high-risk wounds. Larger population studies involving the use of BTM as salvage reconstruction would be beneficial.

## Declaration of competing interest

N/A.
